# Efficiency and Humanism: The Impact of the Humanistic Charting Tool on Patient Experience in the Emergency Department

**DOI:** 10.5811/westjem.62128

**Published:** 2026-08-29

**Authors:** Namrata Garg, Omid Boozarpour, Jose Alaras, Ivy Tran, Christopher R. Peabody, Nicholas Stark

**Affiliations:** * University of California San Francisco, Department of Emergency Medicine, San Francisco, California; † University of California, San Francisco School of Medicine, San Francisco, California

## Abstract

**Introduction:**

Emphasis on documentation challenges clinicians to strike a balance between efficiency and individualized care, often leading to the depersonalization of patient interactions. The Humanistic Charting Tool (HCT) addresses this by integrating the patient’s voice into the electronic medical record (EHR). The objective of this study was to examine whether use of the HCT during emergency department (ED) visits is feasible and improves key patient experience metrics.

**Methods:**

The study took place in an urban, academic hospital. Patients in the ED completed the HCT while waiting to see a clinician. The patient’s responses were condensed into a two-page summary and uploaded into their EHR. Clinicians were asked whether they used the HCT for each patient at the end of the encounter, and patients completed precare and postcare surveys assessing key experience metrics. Our primary outcome measure was changes in patient experience metrics, including key Hospital Consumer Assessment of Healthcare Providers and Systems (HCAHPS) metrics. Our secondary outcome measure was subgroup analyses among patients facing barriers to care.

**Results:**

Approximately 2.8% of the total ED patients were enrolled during the study period. Overall, 129 patients filled out the HCT, and 97 (74%) patients had complete survey data. A total of 65 clinicians participated in the study including 32 attending physicians, 23 resident physicians, and 10 advanced practice practitioners (APP). Clinicians used the HCT for 51 (53%) of these patients. Patients demonstrated significant improvements in feeling that clinicians listened carefully (79% to 92%; *P* < .001), took their preferences into account (61% to 74%; *P* = .01), treated them with courtesy and respect (85% to 94%; *P* < .001), and knew important information about them (43% to 52%; *P* = .01). Patients who did not have a regular clinician (n = 25 [26%]) had a significantly greater increase in feeling involved in their treatment discussion (36% to 60% vs 59% to 66%; *P* = .05). Patients who endorsed at least one social need (n = 15 [16%]) had a significantly greater increase in being listened to carefully (53% to 73% vs 84% to 95%; *P* = .01). No statistical significance was found between patients whose clinicians used the HCT versus not.

**Conclusion:**

The Humanisticc Charting Tool demonstrated feasibility and was associated with improvements in patient experience within the ED. This tool has the potential for patients to share their narratives and enrich their own healthcare experience, which is invaluable in a high-stakes, time-sensitive environment. The results also highlight a novel finding that the HCT may help mitigate disparities in communication, trust, and engagement that disproportionately affect vulnerable populations. This study supports the further development of humanism-centered tools within healthcare systems.

## INTRODUCTION

### Background

The standardization of patient care through electronic health records (EHR) has enhanced productivity and quality of care in various settings. 1–4 However, its emphasis on documentation also challenges clinicians to strike a balance between efficiency and individualized care, often leading to the depersonalization of patient interactions. Documentation time has increased four to five fold with EHR use, significantly reducing the time physicians are able to spend with patients. 5 Clinical standardization within EHRs promotes a more bureaucratic and less individualized approach to patient management. 6 Such depersonalization can hinder the development of patient-clinician rapport, patient satisfaction, and patient-centered care plans that are crucial for improving care.

### Importance

Patient experience is increasingly recognized as a critical component of healthcare quality, influencing everything from clinical outcomes to adherence to treatment plans to reimbursement. 7,8 In the emergency department (ED), where encounters are often brief and high pressure, the challenge of maintaining humanistic, patient-centered care is particularly pronounced, which can ultimately undermine the patient’s experience of the visit. Understanding the impact of tools that facilitate individualized communication and support the integration of patients’ values and narratives into care planning have the potential to advance healthcare delivery and improve patient experience.

### Goals of this Investigation

The Humanistic Charting Tool (HCT) is a patient-driven questionnaire designed to refocus and individualize the acute care experience based on the needs of each unique patient. The HCT asks open-ended and closed-ended questions about a patient’s life, values, and preferences, and maps the responses onto a standardized, condensed document. The feasibility of the HCT was demonstrated within a lower acuity zone of an ED in a pilot study completed between February and April 2023. 9 In this subsequent study, we aimed to investigate the feasibility and early adoption of implementing the HCT across the entire ED and to explore its association with patient experience outcomes.

## METHODS

### Study Design and Setting

The pilot was conducted at a large urban academic ED and featured a mixed-method analysis of patient and clinician experiences with the HCT. Ethics approval was granted by the institutional review board (IRB #22-37455).

### Selection of Participants

The study included both clinicians and patients. Attending physicians, resident physicians, and advanced practice practitioners (APP) working in the ED between November to December 2024 opted in to participate. Patients eligible for this study were those over the age of 18 who presented to the UCSF ED and were triaged as an Emergency Severity Index 3, 4, or 5. Psychiatric patients and patients who required immediate medical care were excluded. Written informed consent was obtained from all participants.

**Population Health Research Capsule**

What do we already know about this issue?

*Standardization improves efficiency but has also been shown to depersonalize care. Clinicians struggle to strike a balance between the two*.

What was the research question?

*We investigated whether using the Humanistic Charting Tool (HCT) is feasible and improves patient experience in the emergency department*.

What was the major finding of the study?

*Using the HCT led to improvement in feeling listened to (79% to 92%; P < .001) and respected (85% to 94%; P < .001)*.

How does this improve population health?

*Humanism-centered tools have the potential to meaningfully improve patient experience in acute-care settings, especially for those who face barriers to care*.

### Implementation of the HCT

Between November and December 2024, patients waiting for a clinician in the ED were invited to complete the HCT (Figure 1) on REDCap (version 15.5.26, Vanderbilt University) with the help of medical student researchers. The patient’s responses were condensed into a two-page, visually structured summary (Appendix 1) and uploaded into their EHR. Clinicians assigned to the patient were encouraged to review their responses, and clinician use of this information was recorded at the end of the patient encounter. The intervention was deemed complete if the patient completed the required components of the HCT questionnaire, and if the clinician’s use of the responses was recorded.

### Measurements

Prior to completing the HCT, patients were administered a precare survey that inquired about their recent healthcare experiences. Following their ED encounter, patients completed a postcare survey either via phone or in-person. Each patient was assigned unique precare and postcare survey link in REDCap to anonymize and match their precare and postcare survey responses. A copy of the surveys can be viewed in Appendix 2 and 3.

### Outcomes

The primary outcome studied was patient experience metrics after using the HCT, including key Hospital Consumer Assessment of Healthcare Providers and Systems (HCAHPS) metrics for physicians, such as courtesy, taking time to listen, and inclusion in treatment decisions. 10 Secondary outcomes included subgroup analysis of experience metrics by clinician use and barriers to care, as well as user experience of the tool.

### Analysis

A priori sample size calculation was conducted for analysis of variance (ANOVA) comparing the two groups relating to the primary outcome (patient experience metric scores before and after HCT administration). Assuming a large effect size (Cohen *f* = 0.40), an alpha of 0.05, and 80% power, a total sample size of 52 participants (26 per group) was required. We compared average scores for each question across different groups using ANOVA. For the primary analysis, ANOVA was used to compare precare and postcare survey results, as well as the difference based on whether the HCT was used by the clinician. In the secondary analysis, scores were compared across patient subgroups who were identified as having barriers to care.

## RESULTS

### Characteristics of Study Subjects

Approximately 2.8% of the total patients who visited the ED during the study period were enrolled. In total, 129 patients filled out the HCT and 96 patients (74%) had complete presurvey and postsurvey data. Clinicians used the HCT for 51 (53%) of these patients. Of the total participants, 25 (26%) did not see a regular clinician, 17 (18%) reported previously having difficulty following a treatment plan, and 15 (16%) indicated having at least one social need of the following: support; housing; transportation; finances; and resources of daily living. A total of 65 clinicians participated in the study including 32 attending physicians, 23 resident physicians, and 10 APPs.

### Main Results

Patient feedback indicated that 57 (59%) felt their clinician did not already know most of the information collected in the HCT. In addition, 74 (77%) agreed with the importance of their healthcare team having access to the information provided on the HCT; 84 (88%) found the HCT understandable and easy to fill out; and 54 (56%) reported they would continue using and updating the tool in the future (Figure 3). Most patients 73 (76%) expressed a favorable intention to recommend the tool to others.

Patients who completed the HCT experienced a statistically significant increase in key patient experience domains when compared to their prior healthcare encounter: their clinician taking their preferences into account (61% to 74%; *P* = .01); listening to them carefully (79% to 92%; *P* < .001); treating them with courtesy and respect (85% to 94%; *P* < .001); and knowing important information about them (43% to 52%; *P* = .01) (Figure 4a). Patients whose clinician used the HCT demonstrated a greater absolute increase in every experience metric besides feeling involved in their treatment discussion compared to patients whose clinician did not use the HCT, although there was no statistical significance (Figure 4b).

Qualitative patient feedback from the postcare survey reinforced the quantitative findings. Many patients described feeling heard and valued during their encounters, with comments such as, “[The clinician] took my input into account,” and “[The clinicians] were the best—they really listened to me.” Others highlighted improved communication, noting, “Everybody was honest and direct…I appreciated the conversations [the clinician] had with me,” and “My doctor even sat down and talked through the survey…the experience was great.”

In the subgroup analysis, patients who experienced barriers to healthcare—such as those who did not have a regular clinician, previously had difficulty following a treatment plan, or endorsed social needs— demonstrated a larger increase in many of their experience domains compared to patients who did not report barriers (Figure 5). For example, patients who did not have a regular clinician had a significantly greater increase in feeling involved in their treatment discussion (36% to 60% vs 59% to 66%; *P* = .05). Similarly, patients who endorsed at least one social need had a significantly greater increase in being listened to carefully (53% to 73% vs 84% to 95%; *P* = .01). While not statistically significant, patients with difficulty following a treatment plan had a greater increase in feeling involved in their treatment discussion (47% to 65% vs 54% to 64%; *P* = .05), and patients who endorsed at least one social need had a greater increase in reporting being treated with respect (53% to 87% vs 91% to 95%; *P* = .13).[Fig f2-wjem-27-1499]

## DISCUSSION

In the fast-paced and time-sensitive environment of the ED, patient encounters often prioritize efficiency and standardized protocols. Within this context, clinicians frequently struggle to balance these demands with the need to provide humanistic, individualized care, and the patient experience often suffers. The HCT represents one approach designed to help clinicians bridge this gap by supporting more personalized interactions and tailoring care to patient needs. Ultimately, our study demonstrates how such interventions are feasible in the ED and have the potential to enhance the patient experience, while also highlighting the practical challenges of implementing patient-centered tools within complex clinical workflows.

The results indicate that patients who filled out the HCT experienced a statistically significant improvement in their self-reported experience metrics. Although we did not explore outcome metrics, previous research strongly links patient experience with improved clinical effectiveness and outcomes. 11,12 These findings underscore the potential value of tools such as the HCT in the ED setting, where time pressures and fragmented encounters often limit opportunities for patient engagement. By enhancing patients’ sense of being heard and involved in their care, such tools may support greater adherence to treatment plans and ultimately contribute to improved health outcomes.

Our study, however, found that this difference in experience was not significant when comparing patients whose clinicians used the HCT with those whose clinicians did not. This suggests that completing the questionnaire may make patients feel more heard, regardless of whether their clinician reviews the responses. One possible explanation for this finding is that completing a questionnaire prompts patients to reflect on their concerns and expectations and may prime them for the encounter by helping them articulate their needs more clearly. Another possible explanation is that clinicians in this study may have altered their behavior during the study period, as they were aware they were being observed and had been primed during study implementation to focus on humanism and patient experience.

The lack of statistical significance with clinical use makes it crucial to examine whether clinicians can realistically engage with the HCT. In fact, the fast-paced nature of the ED may limit their ability to meaningfully integrate the tool into their workflow. A study of patient-based questionnaires in a general medicine clinic showed improved chart completeness and problem-solving without a reduction in total encounter time. 13 It is possible that incorporating a patient questionnaire requires more time or cognitive bandwidth than emergency clinicians can reasonably accommodate if it does not reduce the total encounter time, which may explain why similar benefits were not observed in this setting. Although prior studies have noted positive feedback from both ED patients and clinicians regarding such interventions, 14 evidence evaluating their measurable effects in acute care settings remains limited. Achieving comparable benefits in the ED may require a more streamlined or condensed version of the tool that better fits the rapid workflow and competing demands of emergency care.

Although the use of the HCT by the clinician did not yield statistically measurable benefits in this study, this finding should not discourage EDs from pursuing strategies that promote humanistic, patient-centered care. Notably, patients who reported barriers to care—such as not having a regular clinician, difficulty following a treatment plan, or a social need—demonstrated greater improvements in their experience metrics. This pattern, which has not been previously described in the literature, suggests that tools like the HCT may help “level the playing field” and bring patients facing barriers in healthcare to a more equitable level with their peers. By prompting patients to articulate their needs and enabling clinicians to better understand those needs, such tools may help mitigate disparities in communication, trust, and engagement that disproportionately affect vulnerable populations. In doing so, the HCT has the potential both to enhance individual encounters and contribute to broader efforts aimed at reducing inequities in healthcare delivery.

An innovation such as the HCT represents a low-cost intervention with high impact. The tool is an open-source resource that does not incur direct licensing costs. The primary financial investment is associated with initial integration into clinical workflows and the EHR, which we estimate to require a one-time cost of 100 cumulative hours, or $10,000 of combined administrative and clinician time. Ongoing costs are expected to be minimal, consisting primarily of routine maintenance and periodic staff education, estimated at approximately 20 hours, or $2,000 annually.

Future studies should explore implementation strategies that optimize real-time use of patient questionnaires into the clinician workflow, evaluate their impact on clinical outcomes, and examine their utility across diverse emergency care environments. Special attention should be given to the differential effects of such tools on patients who face barriers and how interventions such as the HCT may influence equity within the ED.

## LIMITATIONS

Several limitations impact the interpretation of these results. First, the study was performed at a single site and only included participants willing and able to use the HCT, limiting the generalizability of the findings. Second, clinicians were aware of the study and may have modified their behavior in response to being observed. Simultaneously, clinicians with a special interest in humanistic patient care may have used their patients’ HCT more effectively in the ED encounter, leading to an inherent selection bias. Additionally, the study did not measure clinical outcomes, limiting our ability to determine whether improvements in patient experience translate into measurable health benefits. The sample size may also have been insufficient to detect smaller effect sizes, particularly when comparing the various subgroups. Future studies with larger sample sizes are needed to more definitively evaluate the effectiveness of the HCT and to confirm the generalizability of these findings.

## CONCLUSION

By honoring each patient’s unique reality and values, the HCT embodies the philosophy of humanism and individualized care. This study demonstrates that the implementation of the HCT is feasible in the ED and suggests a potential to meaningfully improve patient experience, especially for those who face barriers to care. These findings underscore the potential of targeted interventions to strengthen communication, advance equity, and promote patient-centered care within the constraints of acute care settings.

## Supplementary Information



## Figures and Tables

**Figure 1:**
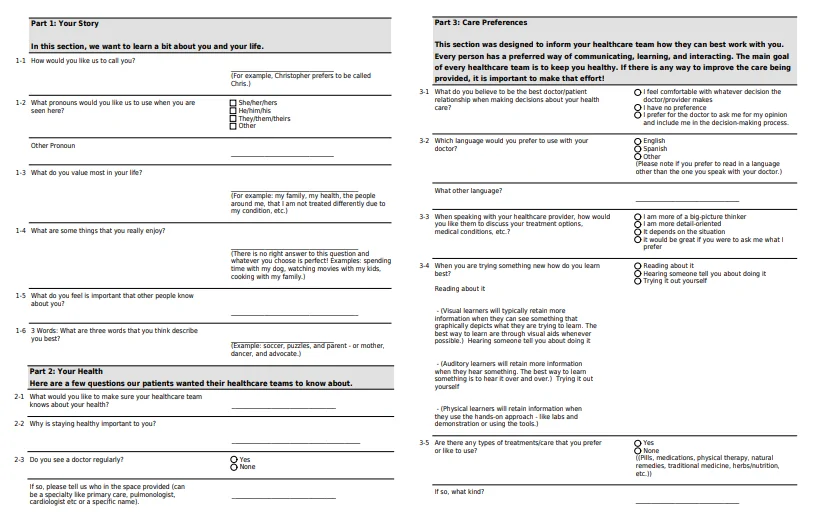

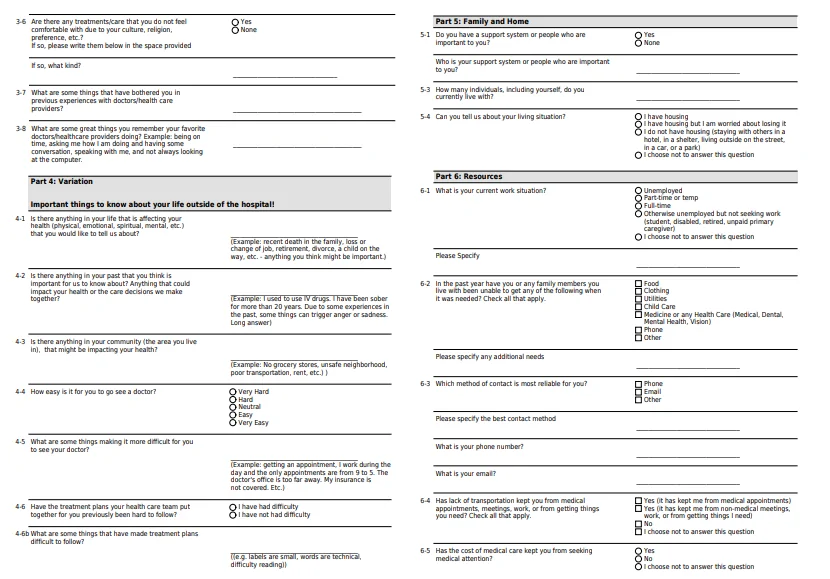
Template of the Humanistic Charting Tool used in a study to examine whether its use during emergency department visits is feasible and improves key patient experience metrics.

**Figure 2 f2-wjem-27-1499:**
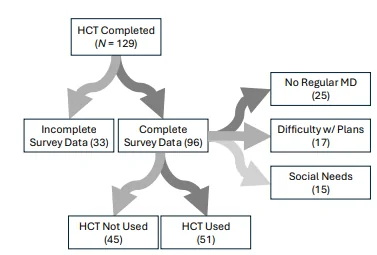
Participant flow chart for a study investigating the impact of the Humanistic Charting Tool. *HCT*, Humanistic Charting Tool; *MD*, medical doctor.

**Figure 3:**
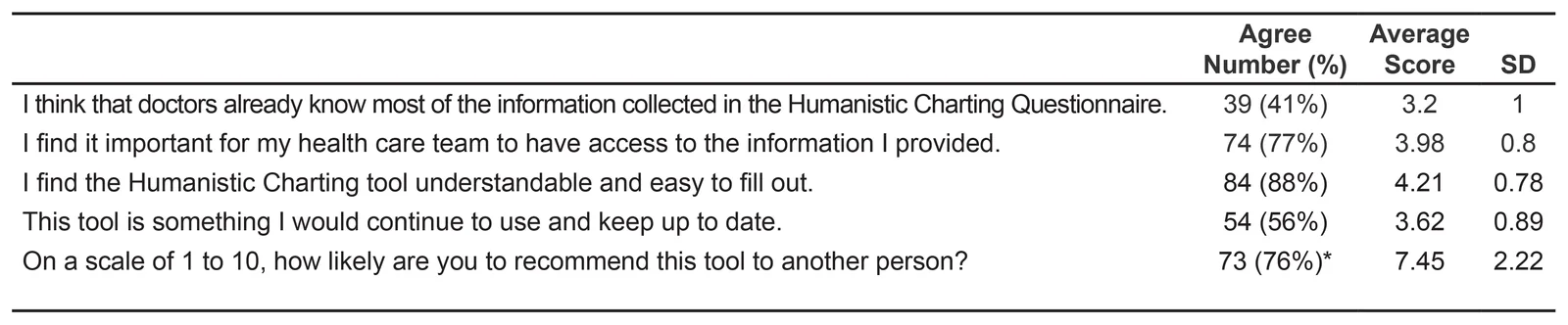
Patient attitudes towards the Humanistic Charting Tool in a study of its impact on patient experience *For this question, agree is considered to be a score of 7 or higher. *SD*, standard deviation.

**Figure 4:**
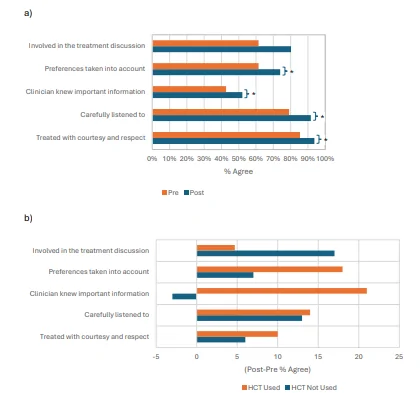
Figure 4a. Comparative analysis of patient experience preintervention and postintervention in a study on the impact of the Humanistic Charing Tool (HCT) on emergency department visits. Figure 4b. Comparative analysis of the change in patient experience preintervention and postintervention when the HCT was used by their clinician in a study on its impact on emergency department visits. *Denotes statistical significance of *P* < .05.

**Figure 5:**
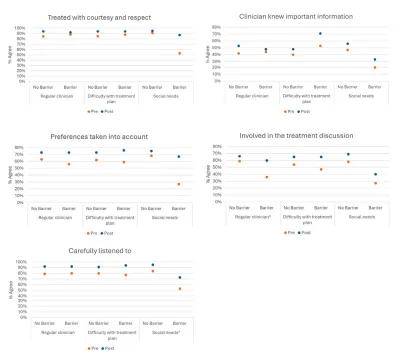
Subgroup analysis of patient experience preintervention and postintervention based on barriers to care in a study on the impact of the Humanistic Charting Tool on emergency department visits. *Denotes statistical significance of *P* < .05.
