# Supplementary material for: Efficiency and Humanism: The Impact of the Humanistic Charting Tool on Patient Experience in the Emergency Department
Source: West J Emerg Med. 2026 Aug 29;27(5):1499–505. doi: 10.5811/westjem.62128 (PMC13660619; doi:10.5811/westjem.62128)
Supplement: Supplementary file 1 [file wjem-27-1499-Appendix.docx]

Appendix 1: Example Copy of the Patient’s Completed HCT (Clinician View)

Appendix 2: Blank Copy of Patient Pre-Care RedCAP Survey

Appendix 3: Blank Copy of Patient Post-Care RedCAP Survey
